# Advances in IoT, AI, and Sensor-Based Technologies for Disease Treatment, Health Promotion, Successful Ageing, and Ageing Well

**DOI:** 10.3390/s25196207

**Published:** 2025-10-07

**Authors:** Yuzhou Qian, Keng Leng Siau

**Affiliations:** 1 Department of Information Systems, City University of Hong Kong, Tat Chee Avenue, Kowloon, Hong Kong, China; yuzhoqian2-c@my.cityu.edu.hk; 2 School of Computing and Information Systems, Singapore Management University, 81 Victoria Street, Singapore 188065, Singapore

**Keywords:** Internet of Things (IoT), artificial intelligence (AI), Internet of Healthcare Things (IoHT), Artificial Intelligence of Things (AIoT), successful ageing, active ageing, age well, sensor-based technologies

## Abstract

**Highlights:**

**What are the main findings?**

- IoT- and AI-integrated healthcare systems enable continuous health monitoring, personalized treatments, and proactive medical interventions for older adults.
- The paper identifies key challenges in privacy, security, ethics, interoperability, and user adoption and proposes multi-level defense mechanisms to enhance system reliability and trust.

**What is the implication of the main finding?**

- Integrating IoT with AI can transform ageing care, improving disease management and promoting healthy, active ageing.
- Future healthcare systems can become more adaptive, patient-centered, and ethically accountable through advancements such as explainable AI, digital twins, and multimodal sensor fusion.

**Abstract:**

Recent advancements in the Internet of Things (IoT) and artificial intelligence (AI) are unlocking transformative opportunities across society. One of the most critical challenges addressed by these technologies is the ageing population, which presents mounting concerns for healthcare systems and quality of life worldwide. By supporting continuous monitoring, personal care, and data-driven decision-making, IoT and AI are shifting healthcare delivery from a reactive approach to a proactive one. This paper presents a comprehensive overview of IoT-based systems with a particular focus on the Internet of Healthcare Things (IoHT) and their integration with AI, referred to as the Artificial Intelligence of Things (AIoT). We illustrate the operating procedures of IoHT systems in detail. We highlight their applications in disease management, health promotion, and active ageing. Key enabling technologies, including cloud computing, edge computing architectures, machine learning, and smart sensors, are examined in relation to continuous health monitoring, personalized interventions, and predictive decision support. This paper also indicates potential challenges that IoHT systems face, including data privacy, ethical concerns, and technology transition and aversion, and it reviews corresponding defense mechanisms from perception, policy, and technology levels. Future research directions are discussed, including explainable AI, digital twins, metaverse applications, and multimodal sensor fusion. By integrating IoT and AI, these systems offer the potential to support more adaptive and human-centered healthcare delivery, ultimately improving treatment outcomes and supporting healthy ageing.

## 1. Introduction

### 1.1. Research Background

The rapid advancement of the Internet of Things (IoT) has driven widespread exploration of its applications across diverse domains, including manufacturing, agriculture, transportation, and healthcare. Increasingly, IoT is being integrated with artificial intelligence (AI), a convergence often referred to as the Artificial Intelligence of Things (AIoT), to achieve more profound and transformative societal impacts.

The Internet of Healthcare Things (IoHT), also referred to as the Internet of Medical Things (IoMT), is a sub-domain of IoT that encompasses uniquely identifiable medical devices connected to the Internet, enabling them to communicate and exchange data [1]. This integration aligns with the shift from Industry 4.0, which emphasizes collaboration between humans and machines, to Industry 5.0, which places humans at the center of technological development. Within this context, the concept of well-being, ageing, and health (WBAH) emerges as a critical concern. WBAH encompasses multiple dimensions, physical, environmental, social, and cultural, that collectively influence the overall quality of life [2,3]. This study addresses the following pressing question: how can the health and well-being of older adults be safeguarded in an era of rapid technological innovation?

The World Health Organization (WHO) places particular emphasis on the ageing population, especially those aged 65 and above, and is making efforts to maintain their physical and psychological well-being. Healthy ageing is described as the process of developing and maintaining functional abilities that enable well-being in later life, including mobility, autonomy in decision-making, relationship building, and contributing to society [4]. However, the demographic shift poses formidable challenges [5]. It is projected that by 2050, one in six people will be over 65 years old, compared to one in eleven in 2019 [6]. Longer life expectancy is accompanied by a higher prevalence of chronic diseases such as diabetes, hypertension, dementia, and Alzheimer’s [7]. Elderly care is further complicated by reduced mobility, cognitive decline, medication management difficulties, social isolation, and sensory impairments [8]. Many of these issues are long-term and cannot be addressed through one-time treatments. Meanwhile, the shrinking ratio of working-age individuals to older adults is creating shortages of healthcare personnel, making it increasingly difficult to meet growing care demands [9].

**Figure 1:**
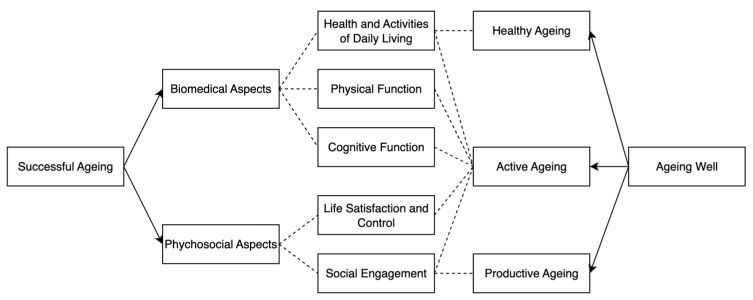
Concepts and Dimensions Related to Successful Ageing.

Meeting only the basic requirement of living is no longer sufficient in the era of Industry 5.0. Instead, society is increasingly focused on promoting and achieving successful ageing. Concepts such as successful ageing and ageing well are multidimensional. Figure 1 (adapted from [10,11]) illustrates the links between ageing concepts and their dimensions. Successful ageing is shaped by both biomedical aspects, covering daily living activities and physical and cognitive functions, and psychosocial aspects, including life satisfaction, control, and social engagement. Ageing well comprises healthy, active, and productive ageing, with the latter emphasizing social participation. Together, these dimensions highlight that both health and psychosocial well-being are essential pathways to ageing well [10]. The ageing population requires particular attention and, therefore, has caught the eyes of different authorities around the globe. For instance, the Singapore government has launched initiatives such as the Age Well Neighbourhoods initiative, which is constructing community care apartments to support the achievement of successful and happy ageing. Several modern technologies, such as alarm systems and advanced monitoring systems, are equipped in such senior-friendly neighborhoods. It can be anticipated that more integration of modern technologies will be needed to provide further assistance to the ageing population.

To address these challenges, assisted living (AL) and healthcare monitoring (HM) solutions are becoming indispensable, requiring continuous and remote care [12]. IoT-enabled systems offer a foundation for continuous and remote care, transforming traditional care environments into smarter ecosystems where health conditions and daily activities can be continuously monitored and managed [8]. At the core of these systems are wireless sensor networks (WSNs), which support continuous health and activity monitoring through wearable, implantable, and ambient sensors [13]. These networks enable real-time data collection and proactive interventions. When combined with AI algorithms, they allow anomaly detection, health risk prediction, and the delivery of timely alerts to caregivers.

Recent advances in IoT, AI, and big data analytics have further accelerated the development of intelligent healthcare platforms. Technology leaders such as Amazon (AWS IoT Core, Washington, US), Microsoft (Azure IoT Hub, Washington, US), Google (Cloud IoT Core, California, US), and IBM (Watson IoT Platform, Munich, Germany) are deploying solutions that enhance device interoperability, streamline data processing, improve disease management, optimize hospital operations, and strengthen patient engagement [14]. Together, these innovations highlight the transformative potential of IoT and AI in healthcare, particularly in addressing the pressing challenges of ageing populations.

Despite these developments, the potential benefits for elderly populations remain underexplored. Few studies address their unique needs, such as simplified usability and the management of conditions like loneliness and mental health disorders. In response to these challenges and opportunities, this study provides an overview of how advances in IoT, AI, and sensor-based technologies can be leveraged to support disease treatment, health promotion, successful ageing, and ageing well. This paper can serve as a start for further in-depth discussion on relevant topics.

### 1.2. Methodology

This paper is a narrative review. Review articles generally aim to synthesize and evaluate existing research to indicate the current state of knowledge in a discipline [15]. Different types of reviews serve different purposes. Narrative reviews (often referred to as literature reviews) provide a broad overview of the literature on a topic. Scoping reviews adopt a systematic approach to map key concepts, theories, sources, and knowledge gaps. Systematic reviews address specific research questions using explicit and unbiased methods to gather all evidence that meets predefined eligibility criteria. Other review types include meta-analyses, mixed-method reviews, rapid reviews, and others.

In this study, we adopt a narrative review approach to provide an overview of technologies such as IoT, IoHT, and AI and their applications in healthcare and ageing. Specifically, we synthesize insights on recent technological advancements, their integration with other innovations such as AI, and their role in enhancing the quality of life for older adults and supporting successful ageing. Unlike systematic reviews, which are designed to answer narrowly defined research questions, this narrative review highlights trends, opportunities, and challenges in IoT-based healthcare systems. Moreover, narrative reviews allow more flexibility in identifying and integrating relevant literature across multiple sources, rather than relying on comprehensive and rigid search protocols [15].

The rest of the paper is structured as follows: Section 2 introduces the IoT-based systems and the integration of modern technologies, such as AI. Section 3 discusses how IoT and AI systems are applied in disease treatment, health promotion, and active ageing. Section 4 shows the challenges and corresponding defense mechanisms of implementing IoT and AI-empowered systems into the daily lives of the ageing population. Section 5 presents the expected societal impacts of such systems, future research directions, and limitations of the current study, and it concludes the paper.

## 2. IoT-Based Systems and AI Integration

Technologies increasingly serve to prioritize human values, and the IoT is a prominent example. As a modern technological paradigm, IoT is becoming integral to daily life, offering convenience and improved quality of living. Derived from innovations in information and communication technologies, IoT is characterized by ubiquitous computing and intelligence [16]. It involves three core components: data collection, information processing, and decision-making based on analytical outcomes [3]. The efficiency of IoT is driven by advancements in supporting technologies. Specifically, sensors and other input devices collect data, information is processed in cloud platforms or edge networks, and decision-making is often enhanced by AI [3]. IoT has been widely adopted across various domains, including energy, agriculture, transportation, and smart city development [17]. Its ultimate goal is to provide seamless connectivity, anytime and anywhere, for anyone and anything [18]. Real-world entities such as electronic devices, sensors, physical objects, living beings, and virtual data can be assigned unique digital identities, enabling centralized monitoring and control [18]. In healthcare, IoHT networks focus on linking and sharing data among diverse medical devices and equipment within healthcare ecosystems, thereby enhancing patient care and supporting innovative medical practices [19,20,21]. Typical input devices include wearable health monitors and other context-specific technologies, while the primary users are healthcare professionals such as physicians. Big companies, such as Apple, are promoting wearable devices, such as Watches, that are equipped with health-tracking features, such as fall detection and sleep tracking.

**Figure 2:**
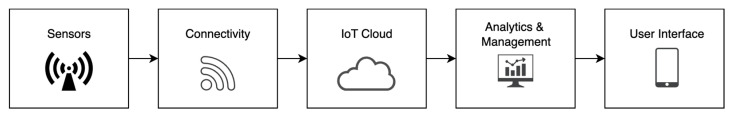
Operating process of IoT systems.

Figure 2 illustrates the operating process of IoT systems. Data collection is enabled by sensors and input devices that gather information from the environment. Information transmission and processing occur via communication protocols, cloud infrastructure, or edge networks. Decision-making and user interaction are often powered by artificial intelligence (AI) and delivered through analytics platforms or user interfaces. The following sections provide an overview of each stage of this process.

### 2.1. Stage 1: Data Collection (Perception Layer)

Advancements in IoT are driven by continuous technological innovations, including WSNs and nanotechnology [22]. At this initial stage, data is captured through a variety of devices, such as audio and video-based systems, as well as specialized sensors embedded in the environment [12]. The IoT architecture relies heavily on different types of technologies, including optical cameras, temperature sensors, biometric identification tools, and other specialized devices [23]. Sensors may be wearable, ambient, or implantable. For example, wearable body area sensors are commonly used for older adults to monitor vital signs and detect health-related events. These sensors capture direct physiological data (e.g., heart rate, glucose levels) as well as indirect behavioral data (e.g., movement patterns) [14].

Some sensors and input devices are specifically designed for medical contexts to facilitate the development of IoHT and IoMT systems. Examples include pacemakers, cochlear implants, and insulin pumps, which can be connected to IoT networks to deliver accurate and personalized data inputs. These data streams support advanced analysis and enable more efficient, timely, and tailored medical treatments [1].

**Figure 3:**
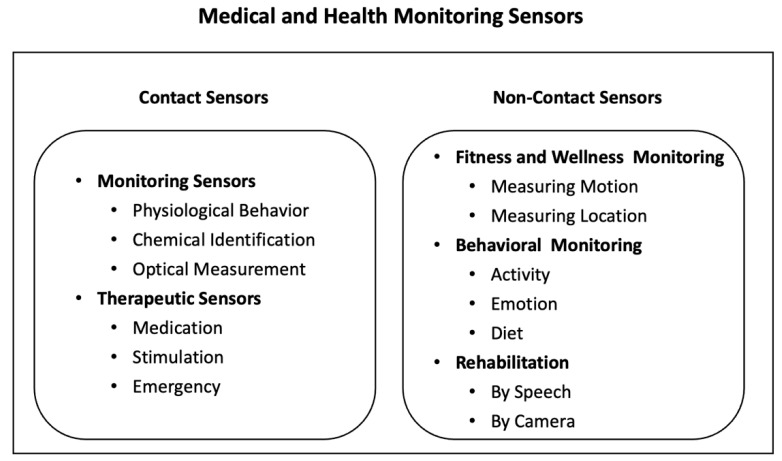
Types of medical and health monitoring sensors [24].

Figure 3 illustrates the classification of medical and health monitoring sensors commonly used in IoT-based healthcare systems. These sensors are broadly categorized as contact or non-contact, supporting applications such as physiological and chemical monitoring, therapeutic interventions, fitness and wellness tracking, behavioral monitoring, and rehabilitation [24]. Such sensors can acquire comprehensive personal health information, providing rich datasets for medical analysis.

It is important to note that data collection in IoT-based systems is not limited to traditional sensors alone; other devices, such as cameras, also contribute significantly to data acquisition. Additionally, actuators play a crucial role in completing the perception layer by performing physical actions in response to analyzed data or automated instructions from cloud-based platforms.

Sensors and devices equipped with advanced AI can now handle more complex pattern recognition tasks, such as hand motion detection with smart gloves, object recognition by robotic manipulators, and lip motion recognition via smart masks [25]. Even traditional sensors for temperature, pressure, and air quality have been enhanced through AI integration, enabling more accurate monitoring and predictive capabilities. The decreasing cost of AI-powered sensors is making IoT devices and systems more affordable and accessible to a broader population [26]. Recent advances in Generative AI (GenAI), which learns data distributions from large datasets and generates new content resembling the training data, are further improving signal identification and encoding [27]. In addition, GenAI-supported virtual health assistants encourage patient involvement, resulting in more data collection [28]. GenAI enhances sensing accuracy and boosts the performance of data collection processes.

### 2.2. Stage 2 Data Transmission (Network Layer)

In this stage, the data collected at the first stage is transmitted either device-to-device or to cloud servers. Reliable and secure connectivity is essential for the effective operation of IoHT systems. To enable real-time, ubiquitous communication between devices and cloud infrastructure, various communication protocols are used [29]. These protocols provide the necessary medium for data exchange and are selected based on factors such as range, bandwidth, and energy efficiency.

Table 1 categorizes commonly used communication protocols based on their effective transmission range [30].

Due to the diversity of protocols used by different devices, gateways play a crucial role in bridging communication gaps. Gateways enable interoperability between heterogeneous devices, translating data from low-level protocols (e.g., Zigbee) into higher-level Internet Protocol (IP) formats. For example, sensor data transmitted via Zigbee can be received by a gateway, encapsulated in IP-based packets, and forwarded through Wi-Fi or cellular networks to the cloud.

Beyond protocol translation, gateways provide several essential functions:

- Seamless communication across heterogeneous networks.
- Data preprocessing to reduce traffic volumes and improve efficiency.
- Security through authentication and encryption.
- Intelligent analytics at the edge, including filtering and early anomaly detection.

**Figure 4:**
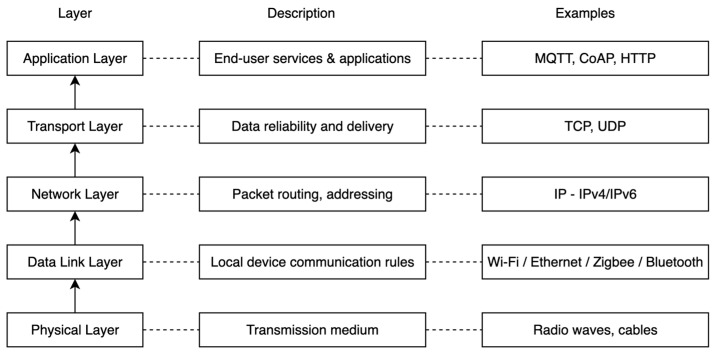
IoT Communication Stack.

Figure 4 illustrates the IoT communication protocol stack, showing the various layers involved in transmitting data from the physical medium to the application level. The stack includes five key layers: physical, data link, network, transport, and application. Each layer is responsible for specific functions, ranging from physical signal transmission to application-level communication.

### 2.3. Stage 3 Data Delivery & Protocol Handling (Transport Layer)

Data delivery at this stage relies on transport layer protocols, such as Transmission Control Protocol (TCP) and User Datagram Protocol (UDP), which ensure that sensor-generated data is reliably transmitted to IoT cloud platforms. At the application layer, IoT-specific protocols, including Hypertext Transfer Protocol (HTTP), Constrained Application Protocol (CoAP), and Message Queue Telemetry Transport (MQTT), are employed to facilitate structured data exchange between IoT devices, cloud services, and end-user applications [31].

The data captured by sensors, devices, and actuators can be processed locally on terminal devices or remotely in cloud environments [32]. To reduce the computational burden on centralized cloud servers and minimize latency, modern IoT systems increasingly adopt decentralized data processing approaches. Processing data closer to the source reduces the need for constant transmission to central servers, thereby improving data transfer rates, decreasing response times, and enhancing overall system efficiency.

Two key paradigms support decentralized processing: fog computing and edge computing.

- Fog computing involves deploying intermediate processing nodes (fog nodes) at the network’s edge, bridging the gap between end devices and the cloud. These nodes handle data computing, storage, and networking tasks with moderate computing capacity and lower latency compared to cloud computing [9].
- Edge computing, on the other hand, installs computing and storage resources directly on end devices or sensors. This approach minimizes latency further by processing data at the source, though it is limited by device-level computing capabilities [9].

Currently, edge processors often suffer from being bulky and power-consuming. AI algorithms address this limitation by enabling new computing architectures that reduce power consumption while keeping devices compact and adaptable [25]. AI has the capability to conduct rapid data processing and interpretation [28].

**Figure 5:**
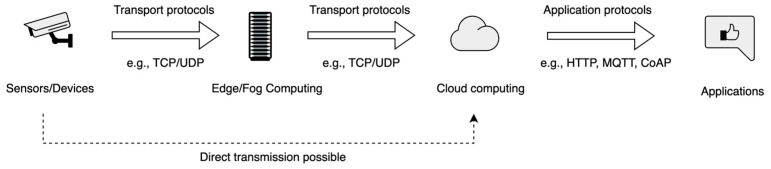
Data delivery and processing architecture in IoT systems.

Based on the discussion, Figure 5 shows the data delivery and processing architecture in IoT systems. Sensors and devices transmit data via transport protocols to edge or fog computing nodes for preliminary analysis. The processed data is then forwarded to cloud computing platforms, where application protocols enable communication with end-user applications. A dashed arrow indicates that direct data transmission from sensors to the cloud is also possible without intermediate edge or fog processing.

### 2.4. Stage 4 Data Processing & Control (Application Layer)

Besides decentralized computing, a lot of data is analyzed on cloud platforms. Specifically, data collected from sensors and other devices is processed on cloud-based platforms, enabling the management of connected devices, aggregation and classification of information, the conduct of analytics, and triggering of alerts or automated actions. Advanced data analytics techniques, including predictive analytics, anomaly detection, and deep learning, are widely applied to process and interpret sensor data, enabling timely medical interventions. GenAI, as a recently advanced technology, for instance, is increasingly being integrated into IoHT-based cloud computing. GenAI’s capacity to synthesize new information from large datasets allows it to streamline resource allocation, refine predictive modeling, and enable healthcare systems to operate in a more adaptive and resilient manner [33,34].

Cloud technologies differ fundamentally from traditional on-premises IT infrastructure in terms of service delivery. Instead of purchasing and maintaining IT assets locally, users can access computing resources owned and managed by cloud providers through internet connections. This service delivery model is referred to as “as a service”, and it is typically categorized into three levels [35].

- Infrastructure as a Service (IaaS): Provides virtualized infrastructure for running applications.
- Platform as a Service (PaaS): Offers tools and platforms for developing and managing applications.
- Software as a Service (SaaS): Delivers ready-to-use application software for end-users.

#### Integrating AI and Deep Learning

The rapid development of AI has significantly enhanced the ability to process vast amounts of healthcare data [36]. AI algorithms support data analysis, decision-making, and real-time response generation, enabling smarter and more efficient IoT-based healthcare systems [7]. AIoT, defined as the combination of AI functions into IoT, performs better in delivering operational efficiency, offering risk management, and providing enhanced services and products [26].

Cloud computing is crucial in healthcare as it enables scalable storage and high-performance processing for large and continuously growing health data volumes [37]. Techniques such as machine learning, deep learning, and predictive modeling can be integrated into cloud platforms to accelerate data analysis and facilitate prompt, informed medical decisions [35].

**Figure 6:**
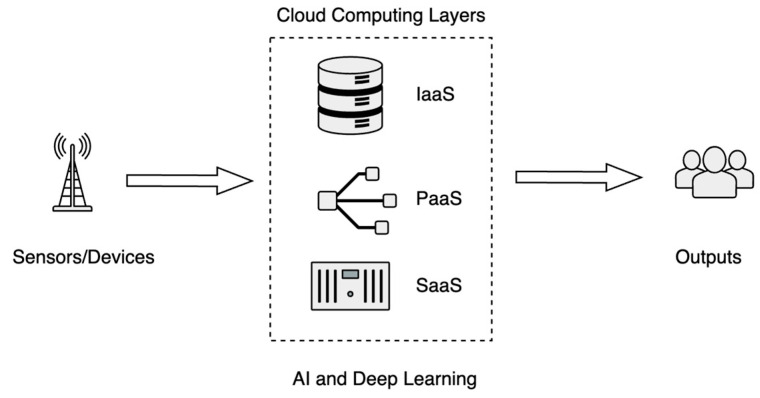
Cloud-based data processing and AI integration in IoT-enabled healthcare systems.

Figure 6 shows how data is processed and controlled with cloud computing and AI integration. Sensor and device data are transmitted to cloud computing platforms comprising three service layers: IaaS for scalable computing and storage, PaaS for application development and management, and SaaS for end-user healthcare applications. AI and deep learning algorithms operate across these layers to enable advanced data analytics. The outputs include actionable insights for healthcare providers, automated alerts, medical decision support, patient monitoring dashboards, and remote device management.

### 2.5. Stage 5 User Interaction & Decision-Making (Business Layer)

At this stage, end-users, including healthcare professionals, caregivers, and older adults, interact with the IoT system through user interfaces such as mobile applications, dashboards, or wearable device displays. These interfaces allow users to monitor health conditions, receive alerts, and send control instructions back to the IoT system to trigger automated responses or manual interventions. Designing intuitive and effective user interfaces is as important as developing robust back-end systems to ensure efficient interaction and decision-making.

Merely monitoring patient conditions is not sufficient to maintain safety and health. It is critical that medical personnel can offer guidance or take necessary actions when intelligent systems fail to make appropriate decisions [38]. In other words, real-time interaction between the IoT system and caregivers is essential. A well-developed IoT architecture should enable seamless, bidirectional communication between clients and connected smart devices [39].

Researchers are actively exploring user interface design improvements to better support caregivers and family members. For example, studies investigate how to integrate visual elements (e.g., graphs, figures), incorporate replay and time-based navigation features, and design emergency alert mechanisms for timely interventions [38,39].

Several key design features and principles have been identified and discussed [40]:

- Usability and Ease of Use

Usability reflects the effectiveness and efficiency of a system in fulfilling user needs, while ease of use determines how smoothly users can operate the system with minimal cognitive load. Both are crucial in healthcare IoT applications. Systems should provide full functionality for both emergency response and long-term health checking. Accessibility is particularly important for older adults unfamiliar with advanced technologies and for caregivers who must make quick, informed decisions. A balance must be maintained between offering multiple functions and preserving a simple, intuitive interface.

- Fault-resistant signaling and alerting systems

Health-related systems need to be designed to minimize user errors that could lead to unintended or harmful outcomes. Interfaces should be intuitive and avoid ambiguous features that might confuse users. Robust and error-resistant alert mechanisms are critical to ensure safety during critical health events.

- Long-term stability

Unlike consumer-grade smart devices with short product cycles, healthcare IoT systems supporting ageing populations often need to operate reliably for many years. These systems must be durable and capable of maintaining full functionality over long periods to ensure uninterrupted patient care.

Successful application design extends beyond these principles. Developers must conduct market research to understand user requirements and continuously adapt their designs to keep pace with evolving technological innovations and healthcare needs.

The application of IoHT is particularly relevant to medical practices. For example, healthcare professionals use IoHT to maintain electronic health records (EHRs), support telemedicine platforms, and enhance clinical decision-making. At the same time, IoHT serves as the foundation for patient-facing applications, such as mobile apps and health portals that allow individuals to monitor and manage their health status [19]. Modern technologies such as GenAI further extend these capabilities by enabling the creation of personalized assistants tailored to individual needs, for instance, sending medication reminders, providing lifestyle recommendations, or facilitating patient–provider communication [28].

## 3. IoT, AI in Healthcare, and Active Ageing

Modern technologies are increasingly integrated to facilitate disease management, health promotion, and active ageing and thus promote the development of IoHT and IoMT [41]. IoHT systems allow the exchange and processing of health-status data by combining IoT devices with modern technologies, such as mobile [42]. Medical services and patients are connected with technical and financial feasibility [43]. Health data can be analyzed by edge, fog, and cloud devices. When combined with AI, IoT can form an Intelligent IoT-based Health System (IIHS). IIHS refers to the incorporation of modern technologies to provide intelligent and predictive capabilities [44]. IIHS can be utilized for health screening, illness early detection, treatment plan selection, and real-time decision support [45]. Practical IoT applications for the ageing population include fall-detection wearables, medication adherence systems, remote health monitoring platforms, and smart-home devices that track daily activities. These systems collect, synthesize, and analyze data from multiple sources, leading to more accurate, timely, and personalized medical decisions and interventions [37]. Expanding the use of IoT technologies can improve healthcare access and quality of life for all individuals, particularly those living in rural or underserved areas [39].

### 3.1. Disease Management

IoT is transforming modern healthcare by enabling continuous health tracking and remote patient management. IoHT interconnects medical devices and systems, enabling more seamless data transmission across healthcare networks. By doing so, it creates opportunities to address persistent challenges such as rising medical costs and limited access to healthcare resources [46]. A notable example of its application is during the Coronavirus Disease 2019 (COVID-19) pandemic, when restricted access to on-site medical services highlighted the value of remote care. IoHT systems were deployed to continuously monitor patients’ health status and support the delivery of appropriate medical interventions without requiring physical hospital visits [47]. Its role in smart healthcare is widely recognized [3]. Managing chronic diseases such as Parkinson’s remains a pressing challenge, consuming substantial medical resources, including hospital beds and staff [35]. IoT-based systems can continuously monitor patient health, reducing hospital visits and improving resource utilization. Social isolation and loneliness are significant issues in ageing populations. IoT systems, such as wearable sensors and connected devices, can enhance social interaction and connectivity among users [16]. Wearable sensors track critical physiological signs, allowing healthcare providers to deliver accurate, timely, and personalized treatments [48]. The integration of personalized sensors and actuators improves treatment precision and therapeutic effectiveness [16].

IoHT systems are expected to generate actionable knowledge from large volumes of patient data, enabling early detection of irregularities and disease prediction [49]. For elderly individuals, particularly those living alone, IoT-based health monitoring systems provide continuous observation of vital signs and behavioral patterns, detecting biological or lifestyle changes that may indicate emerging health issues [50].

Remote monitoring applications include glucose level tracking, heart rate monitoring, and mood or depression assessments [51]. These capabilities keep healthcare providers informed and allow patients to receive timely medical assistance without frequent hospital visits. For mental health conditions that are difficult to anticipate or self-report, IoT systems can infer emotional or psychological states by analyzing physiological data such as heart rate variability and blood pressure [51]. The fully interactive technology helps doctors assess the effectiveness of treatment [52]. Additionally, IoT-based health systems enhance preventive care by enabling early detection of emergencies, identifying long-term health trends, and optimizing hospital visit planning. This proactive approach supports better resource allocation and improves patient outcomes [14].

### 3.2. Health Promotion

Beyond disease treatment, IoT- and AI-based technologies also play an important role in health promotion. Health promotion refers to the ability of individuals to understand, control, and improve their own health by becoming informed, proactive, and accountable in managing their conditions [53]. This is particularly critical for older adults, whose health is generally more fragile than that of younger populations and for whom lifestyle changes can be especially challenging [54].

Governments worldwide are beginning to recognize this need. For instance, South Korea has launched platforms that leverage IoT and AI technologies to enhance healthcare services for the elderly [55]. IoT systems equipped with wearable sensors offer a feasible approach, as they can be seamlessly integrated into the daily routines of older adults without disrupting their lifestyles. These user-friendly devices provide continuous feedback on health conditions, empowering individuals to maintain greater awareness and control over their well-being.

In addition to promoting and enhancing health for individuals, IoT and AI-based technologies benefit the overall condition of global health. IoT systems can play a pivotal role in identifying, monitoring, and mitigating the spread of pandemics such as COVID-19 [56,57]. These systems enable the real-time collection of symptom data from users, such as fever, cough, sore throat, and shortness of breath, to support early detection of suspected COVID-19 cases. During treatment, IoHT systems interconnect medical devices to monitor patient health, thereby improving the efficiency and quality of care [58]. Moreover, IoT technologies facilitate virus tracing, tracking, and spread mitigation, delivering substantial benefits to public health and society as a whole [59].

### 3.3. AI Amplifies the Value of IoT-Collected Data

Emerging technologies such as AI and the metaverse are significantly enhancing the capabilities of IoT in healthcare. Real-time data captured by smart sensors can be transferred to and mirrored within virtual environments, enabling more complex and comprehensive simulations and analyses of physical objects [17].

AI introduces new possibilities for IoT-based systems [60]. By integrating AI, IoT systems gain autonomous decision-making capabilities, reducing the need for constant human intervention [17]. AI-powered algorithms can perform diagnostics, provide decision support to physicians, and process large volumes of sensor data to recommend treatments and generate automated alerts [51]. These functionalities enable continuous assessment and monitoring of elderly individuals and conditions [50]. Additionally, AI can support automated medication dispensing, send reminders for medication adherence, and help schedule physician visits [8].

Machine learning (ML), a subset of AI, has become an essential tool in disease diagnosis and predictive analytics. ML techniques assist in data inference, feature extraction, and intelligent decision-making [61]. Typically, data collected from benchmark datasets and real-time sensors is preprocessed, followed by feature extraction tailored for specific ML models. Classification algorithms then generate predictions or recommended actions, which are subsequently communicated to healthcare professionals (e.g., physicians or nurses) for clinical validation [9].

Robotics is a field that has been significantly enhanced by AI. Robotics is the science that designs, builds, and deploys robots. Robots are programmable and show autonomy while carrying out tasks. Robots enabled by IoT (known as the Internet of Robotics Things—IoRT) connect the capabilities of IoT with robotics and can help perform certain tasks in surgeries and rehabilitation, and notify physicians when unusual signs are detected [62,63].

Beyond decision support, AI enables personalized interventions. Suggestions and actions generated by AI can be tailored to meet individual patient needs. For example:

- Physical assistance: AI-enhanced mobility devices can detect uneven surfaces and adapt to prevent falls, assisting frail individuals in maintaining mobility [6]. Robots, for example, are equipped with human-like automated motion ability [63].
- Cognitive and psychological support: AI-powered virtual assistants can engage older adults in conversations, aid memory retention, and offer customized exercise programs designed to prevent cognitive decline and neurodegenerative disorders [6].
- Social and emotional well-being: AI-driven chatbots and companion robots can assist with daily activities, provide companionship, and help mitigate loneliness, thereby improving mental health in ageing populations.

### 3.4. Ageing Well and Active Ageing

Beyond individuals requiring medical treatment, many older adults experience suboptimal health conditions and social isolation. Loneliness significantly impacts health outcomes, often leading to reduced physical activity and exacerbating negative health effects. This highlights the urgent need to promote active ageing, which encourages older individuals to maintain physical, mental, and social engagement as they age [64].

IoT technologies provide effective tools to support active ageing. By offering customized exercise programs and ensuring seamless integration of heterogeneous devices, IoT systems encourage older adults to engage in regular physical activity [64]. These systems enable real-time health monitoring and analysis, facilitating the creation of personalized care plans tailored to each individual’s needs. Additionally, IoT-enabled platforms foster social engagement by providing communication channels and interactive media that help older adults stay connected with caregivers, family members, and peers. One example of a personalized application is ElliQ, a robot designed to enhance convenience and pleasure in life. It senses environmental and behavioral cues and prompts users proactively, for example, reminding them of taking medications, and suggesting they conduct physical or mental exercises. These decisions are based on individual requirements.

To create more convenient and supportive living environments, researchers and practitioners are developing smart healthcare systems and smart homes. This shift represents a transition from traditional hospital-centric healthcare models to patient- and user-focused care [65]. To create more convenient and supportive living environments, researchers and practitioners are developing smart healthcare systems and smart homes. Smart home health technologies consist of different types, including physiological and safety detection, and social and psychological fulfillment [66]. This shift represents a transition from traditional hospital-centric healthcare models to patient- and user-focused care [61].

Smart homes reduce the demand for traditional healthcare services while improving the quality of life and convenience for older adults. Equipment and furniture commonly applied in smart buildings, such as smart-robotic gripper systems, realize their functions with the assistance of AI sensors [25]. AI-powered analytics can detect behavioral patterns that may otherwise be overlooked and identify hidden relationships between health factors. For example, research has shown that an elderly person’s activity levels can be predicted based on their sleep quality and duration [61]. Companion robots and other digital home devices are becoming typical components of these smart healthcare systems, providing physical assistance, cognitive support, and emotional companionship [38].

A notable area of focus in active ageing research is fall detection and prevention. Falls are one of the leading causes of injury among older adults, with serious consequences including physical harm, reduced mobility, and increased fear of future falls, which can further limit social interactions [61,67]. To improve the accuracy and timeliness of fall detection, researchers are integrating multiple technologies, such as wearable sensors, cloud computing, deep learning, and AI-based data analytics [67]. Wearable devices are particularly valuable as they monitor movement without disrupting daily routines, while AI models enhance the reliability of fall warnings.

Recent advances in machine learning have shifted the focus from traditional post-fall detection to fall prevention strategies. These systems conduct risk assessments and make proactive projections of fall likelihood and direction, enabling timely interventions that significantly reduce fall-related injuries [61].

Wellness is the optimal and ideal condition of health. It indicates the physical, psychological, social, and economic fulfillment of individuals [68]. With the ongoing advancements in IoT, AI, IoHT, and AIoT, an increasing range of applications can be anticipated to enhance the convenience and quality of life for the elderly population.

## 4. Potential Challenges and Concerns

While IoT and AI systems offer promising solutions to address ageing-related issues, their widespread deployment faces several technical, ethical, and societal challenges [69]. To gain public trust and achieve broad adoption, developers must ensure generalizability, transparency, ethical compliance, and accountability. Moreover, IoT-based healthcare systems must be scalable, reliable, and accurate, maintaining consistent performance even as the number of connected devices grows.

Table 2 summarizes key requirements and detailed elements necessary for the successful implementation of IoT-based healthcare systems.

### 4.1. Challenges

Below, we discuss several major challenges in greater detail. These challenges arise whenever IoT and AI systems are deployed for disease treatment, health promotion, or active ageing. While some of these issues have been noted in other contexts, they take on particular significance in healthcare for older adults. This is because seniors are often less familiar with technology-enabled living, and because the devices involved in such systems collect highly sensitive healthcare data continuously over extended periods of time.

#### 4.1.1. Privacy and Security

Data privacy and security remain critical concerns, particularly in medical and healthcare applications [48,70]. Medical data is highly sensitive and is required to maintain confidentiality, availability, and integrity [71]. Improper handling can lead to severe consequences. The integration of AI with IoHT, and potentially agentic AI in the near future, has increased the complexity of healthcare systems. This underscores the importance of proactively addressing individual privacy and data security concerns.

- Data Collection and Maintenance

Wearable sensors and other IoT devices often collect personal data without explicit user awareness. Ensuring compliance with privacy regulations and protecting patient information is a key challenge [37]. Meanwhile, the integration of AI triggers the amount of IoT data. The data may contain irrelevant noise and redundancies if robust AI techniques for preprocessing and preparing data are missing [26].

- Authentication and Integrity

Strong authentication and access control mechanisms are required to prevent unauthorized access to sensitive data. Maintaining data integrity is essential for precise and timely medical diagnoses and treatments [48].

- Cybersecurity Risks

The interconnected nature of IoT devices increases vulnerability to cyberattacks. A single point of failure can affect numerous users simultaneously [72]. For example, cyberattacks on cloud providers can disrupt data hosting and maintenance, damaging trust and hindering adoption [22]. Strict regulations and security standards are therefore essential for data confidentiality and the secure management of patient health information.

#### 4.1.2. Ethical Concerns

One of the key challenges of IoHT is to generate technologically robust and reliable outcomes while remaining ethically responsible, respectful, and trustworthy [73]. Beyond privacy and security concerns, which primarily address technical and operational issues, it is equally important to consider the broader social impacts such systems may create. For example, the widespread adoption of IoHT can reduce the need for patients to visit medical centers, thereby limiting opportunities for personal and social interactions with caregivers [74]. Furthermore, IoHT systems may produce evaluations or recommendations without sufficient contextualization. Unlike traditional medical treatment, which relies on clinical tests and verbal communication, IoHT-driven care risks overlooking patients’ social, mental, and emotional conditions [73].

#### 4.1.3. Technology Transition

The transition from legacy healthcare systems to IoT-based solutions presents significant challenges. Healthcare institutions require compatibility and flexibility during implementation to avoid operational disruptions [75]. Yet, the transition process is costly and demanding [52].

- Interoperability

The sensors and devices to collect data may be heterogeneous. Integrating devices from multiple vendors often suffers from a lack of standardized protocols, making interoperability difficult [9,16,75].

- Role of Gateways

Gateways play a critical role in enabling seamless communication among heterogeneous devices and ensuring smooth integration into existing systems. Designing and proposing standardized gateways and relevant policies are important for the smooth and safe operation of IoT systems.

#### 4.1.4. Technology Aversion

Many older adults have limited exposure to modern technologies, leading to resistance and low adoption rates. Low digital literacy can cause suspicion, fear, and reluctance to engage with IoT or AI-driven healthcare solutions [64].

- System Complexity

Combining IoT with AI introduces complex operational scenarios, which may intimidate older users [60]. The convenience and ease of use of the systems are crucial to enhance the willingness of older people to adopt IoT- and AI-supported systems to assist in their daily lives.

- Adaptability

To address this challenge, IoT-based healthcare systems must be adaptable and easy to use, ensuring they meet the needs of older adults while minimizing operational complexity [16]. The systems need to recognize and fit into different activities, which demand well-developed technologies and regulations.

### 4.2. Defense Mechanisms

To address the multifaceted challenges associated with IoT- and AI-enabled healthcare systems, especially in terms of privacy and security, a combination of technical, policy, and awareness-driven solutions is necessary. Below, we summarize major defense strategies across different levels.

#### 4.2.1. Perception-Level Solutions

The first and most essential step is to raise awareness about the existence and severity of the problems, such as privacy and security issues, across all stakeholders, developers, managers, policymakers, caregivers, and end users [48]. Without this shared understanding, subsequent interventions may lack coordination or fail to address key vulnerabilities.

- Interdisciplinary Collaboration

The agreement and recognition of the existing problems are fundamental to interdisciplinary collaboration among different parties. The development of smart healthcare systems, particularly for accurate disease management and active ageing, requires cross-disciplinary expertise. For instance, machine learning algorithms used for behavioral analysis benefit significantly from knowledge in cognitive science and neuroscience [12]. This collaborative approach enhances both system performance and trustworthiness.

- Inclusive Design

The design of IoHT systems should be grounded in the perspectives of diverse stakeholders to ensure broad usability and acceptance. This means not only considering the technical priorities of designers and developers but also actively integrating the expectations and needs of end users [76]. Taking this inclusive approach helps to surface ethical concerns early in the design process and ensures that solutions are developed in a way that is both technologically robust and socially responsive. System performance should therefore be assessed through a multidimensional lens, balancing the interests of patients, caregivers, healthcare professionals, and policymakers.

#### 4.2.2. Policy-Level Solutions 

Strong and adaptive governance frameworks are critical [77]. Laws, guidelines, and organizational policies must be designed to balance technological advancement with ethical responsibility, especially in protecting the rights and equity of older adults.

- Legal Infrastructure

Establishing comprehensive legal frameworks is crucial for guiding how IoT and AI systems are developed, deployed, and managed. These should address ethical concerns, data ownership, usage consent, and user autonomy [8]. For example, principles related to storage management and data provisions, including access control and confidentiality, need to be properly established [78].

- Organizational Policies

Internally, institutions should create department-specific transition guidelines to ensure smooth migration from legacy systems to IoT-based platforms. Moreover, given the rapid pace of technological evolution, regulations must remain flexible and adaptive to evolving use cases and system capabilities.

#### 4.2.3. Technology-Level Solutions

Due to the sensitive nature of health-related data, technological safeguards must be comprehensive and robust across the IoT architecture. When an attacker attacks the IoHT system, severe consequences, such as the death of an individual, can occur. Therefore, the IoHT security solutions should be specially set up [71].

- Security Protocols & Regulations

Encryption, secure authentication methods, and compliance with international standards, such as the EU’s General Data Protection Regulation (GDPR) and the US’s Health Insurance Portability and Accountability Act (HIPAA), must be rigorously enforced [39].

- Software & Hardware Protections

It is essential to conduct regular vulnerability assessments and secure all software, firmware, and communication layers. Hardware-level protection, such as encrypted chips or physical isolation mechanisms, can provide an added defense against unauthorized access [23].

- Authentication Innovations and Emerging Security Technologies

Recent advances include cryptography-based and smart gateway-based authentication systems, which are being developed to replace traditional credentials [79]. Meanwhile, techniques like blockchain are gaining attention for enhancing data provenance tracking and secure data sharing [48].

- Adaptive Threat Detection

As attackers increasingly utilize AI to launch sophisticated and stealthy threats, it is critical to train security systems with dynamic and updated data that reflects emerging malware behaviors [23].

- Cloud Security Enforcement

Given the widespread use of cloud computing in IoT systems, stringent data access controls, intrusion detection systems, and encrypted communication channels are necessary to secure cloud-based storage and analytics platforms.

Effective defense against challenges of IoT-based healthcare systems requires a multi-level strategy that integrates awareness-building, policy development, and advanced technical safeguards. These mechanisms must evolve continuously to keep pace with technological advancements and emerging threats.

## 5. Expected Societal Impacts, Limitations, Future Directions, and Conclusions

Advancements in medical and information technologies have contributed significantly to human longevity, making ageing a defining trend in modern societies. This demographic shift has drawn increasing attention from researchers and policymakers, particularly regarding how to ensure a convenient, satisfactory, and dignified life for older adults. As the ageing population expands, greater emphasis is being placed on the importance of successful ageing and ageing well.

Technologies such as the IoT, AI, and emerging concepts like the metaverse hold substantial promise for transforming the care and well-being of the ageing population. These innovations are shifting healthcare from a reactive model, responding only after health issues arise, to a proactive model, where physicians, caregivers, and patients can take early, data-driven actions to prevent adverse health outcomes. The integration of AI-enabled IoT systems has the potential to revolutionize disease treatment, active ageing, and overall healthcare delivery.

Ongoing research is uncovering new opportunities to strengthen healthcare systems through advanced IoT- and AI-enabled approaches. Edge AI brings computational intelligence closer to data sources, reducing latency and enabling rapid decision-making, an especially critical factor in disease treatment and emergency care. Explainable AI (XAI) enhances the transparency and interpretability of AI-generated recommendations, which is critical for fostering trust and adoption while giving older adults a clearer understanding of their health conditions and a stronger sense of control, thereby supporting health promotion. Digital twins and metaverse applications create virtual representations of patients and healthcare environments, allowing complex simulations and personalized care planning [80]. Likewise, multimodal sensor fusion integrates heterogeneous data sources to enhance diagnostic accuracy and system robustness. The immersive environments also expand the possibility for older adults to maintain independence and enrich their daily lives.

The field of healthcare is exploring the emergence of agentic AI systems for the next generation of the “agentic era”. Agentic AI is expected to provide autonomy, adaptability, scalability, and probabilistic decision-making [81]. The discipline is anticipating the agentic AI systems to initiate medical administrative automation, offer surgical assistance, perform drug discovery and development, and come up with personalized treatment.

The societal impact of IoT- and AI-empowered healthcare systems extends beyond medical treatment. AI-based virtual assistants and chatbots are already being widely deployed to assist physicians, provide valuable support to patients, and improve accessibility to healthcare services [39]. These technologies can enhance patient engagement, deliver personalized and adaptive treatments, and bridge healthcare gaps for individuals with mobility challenges or those living in remote and underserved areas [82].

However, realizing these benefits requires robust governance and regulatory frameworks to address data privacy, security, ethics, and accountability in AI-driven IoT systems [60]. Future research should investigate how to design effective policies and governance models that protect sensitive health data while fostering innovation. Another essential avenue is studying strategies to build trust among older adults, which could help overcome digital literacy barriers and reduce psychological resistance to technological adoption [60].

This study presents a review of how IoT, AI, and sensor-based technologies can be deployed to support disease treatment, health promotion, successful ageing, and ageing well. In particular, we discuss the role of IoHT and how it can enhance the quality of life for older adults. As these systems evolve rapidly alongside technological advancements, future research should continue to explore how emerging innovations contribute to building ageing-friendly societies and how older adults perceive and adopt such technologies. Meanwhile, this study does not provide an in-depth discussion of governance frameworks for IoHT and other modern technologies in healthcare. Future work should address this gap by examining regulatory, ethical, and policy mechanisms needed to ensure responsible and sustainable implementation. In addition, building on the insights from this review, future research can formulate more detailed and specific research questions, and subsequent studies may focus on conducting systematic reviews to address them. Methodologically, this study adopts a narrative review approach. Compared with systematic reviews and other structured methodologies, narrative reviews draw from a broader range of sources and follow a less rigorous selection process [83]. As a result, the presentation and synthesis of findings may be influenced by the researcher’s subjective interpretation of the topic [84]. To address these limitations, future studies could undertake more rigorous review designs, applying explicit and well-formulated principles to enhance transparency and reproducibility.

In conclusion, society faces urgent and complex challenges associated with a rapidly ageing population. Maintaining both physical and mental health is crucial for ensuring that the ageing population experiences a fulfilling and successful later life. The convergence of IoT, AI, and emerging digital technologies provides promising, scalable, and transformative solutions for addressing these issues. With ongoing interdisciplinary research, ethical considerations, and sound governance, these technologies can significantly improve disease management, promote healthy ageing, and enhance the overall quality of life for older adults worldwide.

## Figures and Tables

**Table 1:** Communication Protocols based on Range.

Range	Communication Protocols
<100 m	Bluetooth Wi-Fi Zigbee Near Field Communication (NFC) Radio Frequency Identification (RFID) Z-Wave
<5 km	Cellular communication (3G, 4G, 5G)
>5 km	LoRaWAN Sigfox

**Table 2:** Requirements for the successful implementation of IoT-based healthcare systems [16,35].

**Requirements**	**Elements**
Modularity	Heterogeneity Interoperability Maintainability
Availability	Scalability of Technology Reliability Efficiency
Delivery of Service	Scalability of the Seniors Security Wearability
Adaptability	Adaptation Usability Accuracy

## Data Availability

No new data were created or analyzed in this study. Data sharing is not applicable to this article.

## Abbreviations

The following abbreviations are used in this manuscript:

IoT	Internet of Things
AI	Artificial Intelligence
IoHT	Internet of Healthcare Things
IoMT	Internet of Medical Things
AIoT	Artificial Intelligence of Things
WBAH	Well-being, aging, and health
WHO	World Health Organization
AL	Assisted living
HM	Healthcare monitoring
WSNs	Wireless sensor networks
GenAI	Generative AI
NFC	Near Field Communication
RFID	Radio Frequency Identification
IP	Internet Protocol
TCP	Transmission Control Protocol
UDP	User Datagram Protocol
HTTP	Hypertext Transfer Protocol
CoAP	Constrained Application Protocol
MQTT	Message Queue Telemetry Transport
IaaS	Infrastructure as a Service
PaaS	Platform as a Service
SaaS	Software as a Service
EHRs	Electronic Health Records
IIHS	IoT-based intelligent health system
COVID-19	Coronavirus Disease 2019
ML	Machine learning
IoRT	Internet of Robotics Things
IMU	Inertial unit measurement
GDPR	General Data Protection Regulation
HIPAA	Health Insurance Portability and Accountability Act
XAI	Explainable AI
